# Hibernating female big brown bats (*Eptesicus fuscus*) adjust huddling and drinking behaviour, but not arousal frequency, in response to low humidity

**DOI:** 10.1242/jeb.246699

**Published:** 2024-03-07

**Authors:** Kristina A. Muise, Yvonne A. Dzal, Quinn E. Fletcher, Craig K. R. Willis

**Affiliations:** Department of Biology, University of Winnipeg, 515 Portage Ave, Winnipeg, MN, Canada R3B 2E9

**Keywords:** Heterothermy, Huddling behaviour, Evaporative water loss, Hibernation, *Eptesicus fuscus*

## Abstract

Many mammals hibernate during winter, reducing energy expenditure via bouts of torpor. The majority of a hibernator's energy reserves are used to fuel brief, but costly, arousals from torpor. Although arousals likely serve multiple functions, an important one is to restore water stores depleted during torpor. Many hibernating bat species require high humidity, presumably to reduce torpid water loss, but big brown bats (*Eptesicus fuscus*) appear tolerant of a wide humidity range. We tested the hypothesis that hibernating female *E. fuscus* use behavioural flexibility during torpor and arousals to maintain water balance and reduce energy expenditure. We predicted: (1) *E. fuscus* hibernating in dry conditions would exhibit more compact huddles during torpor and drink more frequently than bats in high humidity conditions; and (2) the frequency and duration of torpor bouts and arousals, and thus total loss of body mass would not differ between bats in the two environments. We housed hibernating *E. fuscus* in temperature- and humidity-controlled incubators at 50% or 98% relative humidity (8°C, 110 days). Bats in the dry environment maintained a more compact huddle during torpor and drank more frequently during arousals. Bats in the two environments had a similar number of arousals, but arousal duration was shorter in the dry environment. However, total loss of body mass over hibernation did not differ between treatments, indicating that the two groups used similar amounts of energy. Our results suggest that behavioural flexibility allows hibernating *E. fuscus* to maintain water balance and reduce energy costs across a wide range of hibernation humidities.

## Teaser

**Highlighted Article:** Female big brown bats maintain more compact huddles and increase drinking behaviour when hibernating in a dry environment versus a humid environment. Adjusting hibernation behaviour could allow this species to maintain water balance across a range of humidities.

## INTRODUCTION

During seasonal periods of low ambient temperature (*T*_a_) and food availability (i.e. winter), many mammals use hibernation, characterized by long, multi-day bouts of torpor (Geiser, 2004). During torpor, mammals show a controlled reduction of their body temperature (*T*_b_) set-point, often to within 1–2°C of the surrounding *T*_a_ (Geiser, 2004). This decrease in *T*_b_ is coupled with a dramatic reduction in metabolic rate (MR) that can reduce energy expenditure by up to 99% relative to remaining at normothermic *T*_b_ (Geiser, 2004).

Torpor results in enormous energy savings but mammals cannot stay torpid through the entire hibernation period, and periodically arouse, restoring MR and *T*_b_ to normothermic levels. These periodic arousals are energetically expensive and can account for over 90% of a hibernator's winter energy budget despite representing only ∼1% of a hibernator's time budget (Karpovich et al., 2009; Thomas et al., 1990a). Previous studies have hypothesized that arousals help to restore immune function (Prendergast et al., 2002), repay a sleep deficit (Daan et al., 1991), provide opportunities to forage (Hope and Jones, 2012), restore the balance of metabolites such as ketone bodies or carbohydrates (Baumber et al., 1971; Galster and Morrison, 1970), and provide an opportunity to excrete metabolic waste, which accumulates slowly during torpor (Németh et al., 2010). Another likely function of arousals is to allow hibernators to drink and replenish water lost during torpor (Ben-Hamo et al., 2013; Thomas and Geiser, 1997). Hibernators lose water to the environment either through the respiratory tract (respiratory evaporative water loss, EWL) or across the skin (cutaneous EWL), with the sum of these losses equal to total EWL. During prolonged torpor, respiratory rate is dramatically reduced and may include extended periods of apnoea (Thomas et al., 1990b), and thus rates of respiratory EWL are relatively low. Despite these low rates of EWL, however, water is still lost during torpor and some hibernators appear to arouse more frequently in dry conditions, presumably to drink and restore water balance (Thomas and Geiser, 1997).

Some hibernators huddle together in groups during hibernation. Huddling could allow for social thermoregulation with individuals sharing thermoregulatory costs but could also help reduce EWL by reducing an individual's surface area exposed to ambient conditions (Gilbert et al., 2010). Boratyński et al. (2015) measured MR and EWL in solitary individuals and groups of four or five hibernating Natterer's bats (*Myotis nattereri*) and found that huddling reduced EWL by almost 30% with no change in MR. Boratyński et al. (2015) attributed this reduction in EWL to the decrease in skin exposed to ambient conditions and suggested that the direct benefit of huddling during hibernation was to reduce EWL. However, by reducing EWL, they suggested that huddling could also provide an indirect energetic benefit by reducing the frequency of arousals stimulated by water loss and the need to drink (Boratyński et al., 2015). Although Boratyński et al. (2015) found no direct effect of huddling on energy expenditure during torpor, a reduction in exposed surface area resulting from huddling could also reduce an individual's energetic cost of rewarming (Gilbert et al., 2010). Boyles et al. (2008) provided evidence that a reduction in exposed surface area in huddling Indiana bats (*Myotis sodalis*) resulted in a lower energetic cost for individuals when rewarming to normothermic *T*_b_ due to a reduction in heat loss.

Understanding the relationship between behaviour, EWL and energy expenditure for hibernating bats has become an increasing concern because of white-nose syndrome (WNS), a fungal disease caused by the pathogen *Pseudogymnoascus destructans*. Currently, WNS is causing mass mortality in North American hibernating bats (Cheng et al., 2021; Hoyt et al., 2021). WNS has affected 12 hibernating North American bat species, but impacts vary among species with some showing rapid and severe declines, while others are virtually unaffected (Cheng et al., 2021). WNS increases arousal frequency, and thus energy expenditure during hibernation (Reeder et al., 2012; Warnecke et al., 2012, 2013). The exact cause of the increased arousals is not fully understood, but the ‘dehydration hypothesis’ (Cryan et al., 2010; Willis et al., 2011) posits that damage to the skin of flight membranes by fungal lesions increases EWL, which, in turn, causes bats to increase arousal frequency, and thus increases overwinter energy expenditure. Consistent with this hypothesis, McGuire et al. (2017) found that little brown bats (*Myotis lucifugus*) inoculated with *P. destructans* showed higher levels of EWL, along with increased torpid MR, both of which could lead to increased arousal frequency and greater overwinter energy expenditure.

The impact of WNS on big brown bats (*Eptesicus fuscus*) has been moderate compared with that on other species, and this species appears partially resistant to WNS (Cheng et al., 2021). However, mechanisms underlying resistance are not fully understood (Frank et al., 2014). Moore et al. (2018) found that captive big brown bats inoculated with *P. destructans* expressed longer torpor bouts than sham-inoculated controls, which would translate into greater energy savings for inoculated bats. This suggests that big brown bats adjust thermoregulatory patterns during hibernation in response to WNS, but behavioural adjustments could also explain their apparent resistance to severe infection or an ability to maintain lower *P. destructans* loads in the first place. Big brown bats are not as gregarious during hibernation as some of the more heavily impacted WNS-affected species, but they have been observed huddling in groups of up to ∼30 individuals (Fenton, 1972; Brack and Twente, 1985; Moosman et al., 2017; Phillips, 2014). This species also roosts across a wider range of humidity than species heavily impacted by WNS, including dry environments that inhibit *P. destructans* growth (Klüg-Baerwald and Brigham, 2017). Haase et al. (2019) modelled the effect of humidity on survival of WNS-affected little brown bats and concluded that humid conditions dramatically increase mortality rate because high humidity accelerates *P. destructans* growth. Thus, the ability of big brown bats to roost in drier environments than little brown bats, which are associated with lower *P. destructans* growth, could play a major role in their resistance to WNS (Klüg-Baerwald and Brigham, 2017).

Our objective was to understand how hibernating big brown bats tolerate variation in humidity. We tested the hypothesis that big brown bats use behavioural flexibility during torpor and arousals to maintain water balance and reduce energetic costs throughout hibernation. We predicted that bats hibernating in a dry environment would drink more often during arousals, and form more dense, compact huddles during torpor, but show no difference in the frequency or duration of arousal or torpor bouts and thus no difference in total loss of body mass (a proxy for overwinter energy expenditure), compared with bats in a humid environment.

## MATERIALS AND METHODS

### Ethics statement

All research was approved by the Animal Care Committee at the University of Winnipeg (protocol AE12193).

### Adjustment to the animal facility

To test our hypotheses, we used a pre-existing captive colony of 20 non-reproductive, adult, female, big brown bats, *Eptesicus fuscus* (Beauvois 1796). We used only females for two reasons. First, a colony of females, made up of individuals that were already acclimated to captivity and each other, was available to us. Second, we predicted that potential effects of humidity on hibernation might be most important for female bats because females must rely on fat left over at the end of hibernation to fuel spring reproduction and are, therefore, more energetically constrained than males (Jonasson and Willis, 2011; Czenze et al., 2017).

Originally, all bats were caught in June 2017 from two netting sites 328 km apart: Bismarck, ND, USA (46.76°N, 100.76°W), and Ada, MN, USA (47.30°N, 96.51°W). At capture, each bat was outfitted with up to two coloured, plastic forearm bands on either their right or left forearm. Bats were housed together at North Dakota State University for 28 months prior to our study. During summer, bats were housed outdoors in 2.5×2.5×2.5 m flight cages (as described by Boyer et al., 2020), and in winter, the bats were moved to modified incubators for hibernation (Erin Gillam, North Dakota State University, personal communication).

We transported all big brown bats to the University of Winnipeg (Winnipeg, MB, Canada) on 19 October 2018. Bats were housed in two groups of 10 in temperature-controlled incubators set at 8°C and humidified by wet sponges. Inside each incubator, bats were housed in custom-built nylon mesh cages (modified from Exo Terra^®^ Flexarium/Flextray PT2556, Hagen Inc., Montreal, QC, Canada; 49.5×20.3×38.8 cm and 43.2×26.7×57.2 cm). Both incubators were equipped with infrared (IR) cameras (Hawk Eye Nature Camera, Songbird Garden, Cape Fair, MO, USA) that gave an overhead view of the mesh cages on a video monitoring system (VMAX480 DW-VMAX-16, Digital Watchdog^®^, Cerritos, CA, USA). The adjustment period lasted from 19 October to 27 November 2018 to ensure bats were in good body condition had recovered from transport before hibernation.

On 20 October 2018, we attached temperature-sensitive dataloggers (iButton^®^ DS2422; Maxim Integrated™, Sunnyvale, CA, USA, modified as per Reeder et al., 2012) to each bat directly on their skin between their shoulder blades using a surgical-grade adhesive (Osto-Bond, Montreal Ostomy, Vaudreuil, QC, Canada) to record skin temperatures (*T*_sk_) of individual bats. *T*_sk_ data from this period were not used in any subsequent analysis but they allowed us to confirm that all bats were periodically entering torpor. Dataloggers were coated in a layer of black synthetic rubber (Plasti Dip^®^, Plastic Dip International, Blaine, MN, USA) to protect the circuit board and battery from humidity (Reeder et al., 2012) and attenuate ultrasound which can be emitted by iButtons and potentially disturb bats (Willis et al., 2009). We painted each datalogger with a unique symbol using white correction fluid (Wite-Out^®^, BiC^®^, Toronto, ON, Canada) to identify individual bats in IR videos. Following hibernation, all dataloggers were calibrated against a NIST-traceable mercury thermometer in a water and ethylene glycol mixture inside a temperature-controlled cabinet.

During the 40 day period of adjustment to the facility, we performed health checks every second day for the bats' first week, once every 3–5 days for the next 2 weeks, and once every 7–11 days for the final 2.5 weeks of adjustment. During theses health checks, we measured body mass (±0.1 g) with an electronic balance (Ohaus^®^, CS200, Pine Brook, NJ, USA), and hand-fed each bat up to 40 mealworms (larval *Tenebrio molitor*) supplemented with vitamins and minerals (Barnard and Bowen, 2013). We provided water and an additional 400–450 mealworms *ad libitum* to each cage following each health check.

### Acclimation to humidity treatments and hibernation

On 27 November 2018, all bats were moved into a second set of larger temperature- and humidity-controlled incubators (Environmental Chamber, Model 6041, Caron^®^, Marietta, OH, USA) set at 8°C and 98% relative humidity (RH). The incubators were humidified using a single condensate recirculating system (Condensate Recirculating System CRSY102, Caron^®^). We housed bats in the same groups of 10 as during the acclimation period to minimize stress from disruption of social dynamics. Bats were housed in a second set of custom-built nylon mesh cages (Exo Terra^®^ Flexarium/Flextray PT2556, Hagen Inc.; 91.4×43.2×43.2 cm). In each incubator, IR cameras (Intense IR Dome Camera, HD5941T, Speco Technologies, Amityville, NY, USA) were installed to continuously monitor the bats. One camera was situated on the ceiling of each cage, and a second was oriented laterally pointing towards the drinking dish. Two temperature and RH sensors were placed inside each incubator (HOBO^®^, Onset^®^, Model S-THB-M008, Bourne, MA, USA), one at the top and one at the bottom of the cage.

We provided water *ad libitum* via tubing that passed into the cages, allowing us to refill water dishes without opening the incubator door and disrupting the hibernating bats. The water dishes contained aquarium rocks to allow bats to climb out of the dishes in case they fell in. On 7 December 2018, for a separate study, we removed iButtons using a medical grade adhesive remover (Uni-solve^®^, Smith & Nephew Inc, Mississauga, ON, Canada). We re-attached the iButtons to the bats on 7 December 2018, which were set to record once every 15 min (±0.5°C) for the entire hibernation period. On 14 December 2018, we measured body mass and hand-fed all bats using the same protocol as described above for the adjustment period. We then decreased the RH in one incubator to 50% (i.e. the dry treatment, water vapour pressure 0.57 kPa at 8°C), while the other incubator remained at 98% RH (i.e. the humid treatment, water vapour pressure 1.03 kPa at 8°C). On 18 December 2018 (experimental day 1), we measured body mass and hand-fed the bats up to 40 mealworms, then returned them to their respective incubator for undisturbed hibernation. Food was withheld throughout the experiment to encourage normal hibernation by matching conditions to those in the wild. On 21 March 2019 (experimental day 93), we opened the dry treatment incubator to repair a hole in the mesh cage. The repair took less than 5 min, and we replicated this disturbance in the humid treatment incubator.

On 8 April 2019 (experimental day 110), we removed all bats from hibernation, removed the iButtons, and measured body mass. Six bats had shed their iButtons during hibernation between 15 January 2019 and 2 April 2019. Four iButtons were shed from the humid treatment bats and two were shed from the dry treatment bats. It was possible to pinpoint the days on which the iButtons were shed by reviewing the IR video, and we only used data from periods when an iButton was attached to a bat. In addition, two iButtons on bats in the dry treatment malfunctioned and did not record any data, and, as a result of a programming error, all remaining iButtons stopped recording on 19 January 2019. This reduced *T*_sk_ recordings to 27–31 days (i.e. starting on 18 December 2018 and ending between 15 and 19 January 2019, depending on the iButton) from 10 bats in the dry treatment and 8 bats in the humid treatment. We used these *T*_sk_ data to confirm that the continuously recorded video provided a good approximation of arousals (see ‘Quantifying arousals’, below) during the same time period (see Figs S1 and S2 for Tsk traces). While removing the bats at the end of the experiment (8 April 2019), unfortunately, we discovered that one bat in the dry treatment had got caught in the tape lining the mesh cage during a day when all bats aroused from torpor and were active. After we reviewed the video in detail, we confirmed that this bat died on 11 February 2019, and we retained its *T*_sk_ data up to that point to use for quantifying arousals (see below), but removed its hibernation data from all of our other analyses. We are confident that movements of this bat on or before 11 February, or the presence of this bat after 11 February, did not disturb other bats in the huddle. Bats in both treatments often aroused in a cascade or sequence over the course of several hours and the arousal in the dry treatment on 11 February was typical of this pattern, with no unusual behaviour by other bats. Their arousals and huddling behaviour after 11 February were also unchanged. In general, we saw no evidence that movements by this bat, or any other bat during an arousal, caused disturbance to other individuals in the huddles. For example, normothermic bats often groomed while in the huddle without disrupting torpid ones.

### Quantifying arousals

Most studies that use measurements of *T*_sk_ to quantify torpor bouts during hibernation rely on an arbitrary *T*_sk_ threshold to define the start and end of torpor bouts and arousals (e.g. when *T*_sk_ was 10°C or more below the highest measured *T*_sk_; Audet and Fenton, 1988; Reeder et al., 2012). However, to our knowledge, no study of any heterotherm has attempted to define arousals using behavioural data, validated by *T*_sk_ data to quantify arousal frequency and duration. As our *T*_sk_ dataset was limited in late hibernation because the iButtons failed to record for the entire experimental period, we used our *T*_sk_ dataset from early hibernation to define a *T*_sk_ threshold that reliably quantified arousal frequency and duration. We then compared arousal frequency and duration quantified from the *T*_sk_ threshold and behavioural observations in the IR recordings during the same time period.

#### *T*_sk_ data

**Fig. 1.**
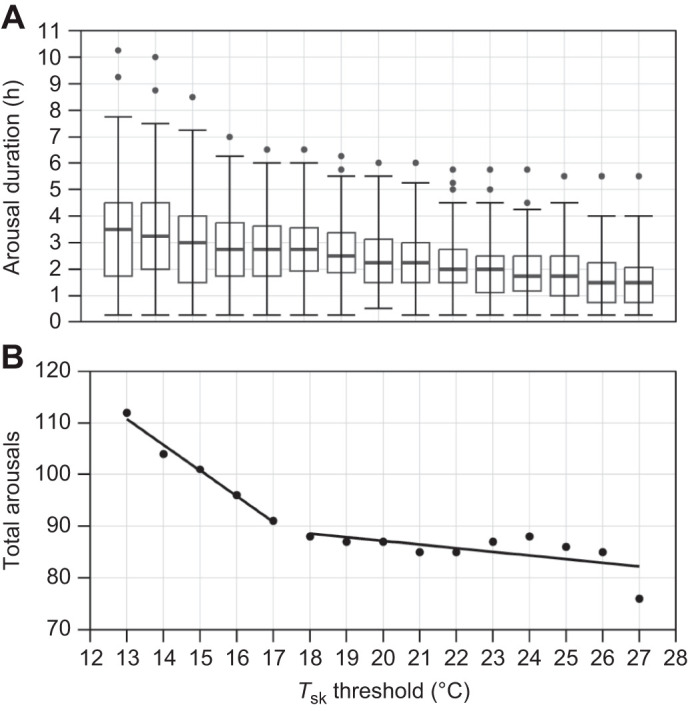
**Defining the skin temperature threshold to quantify arousal duration and arousal frequency in hibernating big brown bats (*Eptesicus fuscus*).** (A) Box plot of arousal duration for each skin temperature (*T*_sk_) threshold we examined from 13°C to 27°C for big brown bats (*n*=18) from both the humid and dry treatments. The median is represented by a solid horizontal line, and the top and bottom of each box represent the 25th and 75th percentiles, respectively. The upper whisker represents the maximum value within 1.5 times the interquartile range above the 75th percentile, whereas the lower whisker represents the minimum value of data within 1.5 times the interquartile range below the 25th percentile. Points that are outside the whisker range are included on the plot. (B) Breakpoint regression showing the relationship between *T*_sk_ threshold and total arousals with a break in the slope occurring at a threshold of 17.4°C.

We used an iterative method to define a *T*_sk_ threshold that best quantified both arousal frequency and arousal duration. We first quantified both the number and duration of arousals for each individual bat using *T*_sk_ thresholds ranging from 13 to 27°C in 1°C increments. We considered bats normothermic if *T*_sk_ increased above the specified *T*_sk_ threshold for at least two datalogger readings (i.e. 15 min) and torpid if *T*_sk_ fell below the *T*_sk_ threshold for at least two readings. We then used linear regression to test for an effect of *T*_sk_ threshold on arousal duration (in h; raw data plotted, but square-root transformed to achieve normality of residuals) (Fig. 1A) and breakpoint regression (R package ‘segmented’; Muggeo, 2017) to test for an effect on the number of arousals detected, followed by linear regression on each segment (Fig. 1B). Not surprisingly, as the *T*_sk_ threshold increased, arousal duration declined (Fig. 1A; *F*_1,1356_=127.8, *P*<0.001, range in number of arousals per *T*_sk_ threshold: *n*=112 for 13°C and *n*=76 for 27°C). However, there was a breakpoint in the effect of *T*_sk_ threshold on the total number of arousals at 17.4°C (Fig. 1B). Below 17.4°C, there was an increase in the number of arousals detected as the *T*_sk_ threshold fell (*F*_1,2_=55.34, *P*=0.02), but above 17.4°C, the relationship between *T*_sk_ threshold and total number of arousals did not differ from zero (*F*_1,8_=4.69, *P*=0.06). This suggests that a *T*_sk_ threshold between 18°C and 27°C provides a reasonable approximation of torpor expression without a significant effect of the *T*_sk_ threshold itself on the total number of arousals.

We then used a repeated measures analysis of variance (RM-ANOVA) (with Bat ID as the repeated factor) (aov; R package ‘stats’; http://www.R-project.org/) to test for an effect of arousal criteria with *T*_sk_ threshold (categorical with 15 levels; i.e. 13–27°C), and one level as behaviour observed in the IR video on arousal duration (square-root transformed to achieve normality of residuals). We used Levene's test for equality of variances among groups (all *P*>0.05). The overall RM-ANOVA model was significant (*F*_1,1429_=37.45, *P*<0.001) so we followed it with Dunnett's *post hoc* test (R package ‘DescTools’; https://cran.r-project.org/package=DescTools) to compare arousal duration quantified with the behavioural measurements against arousal duration quantified through *T*_sk_ measurements at each *T*_sk_ threshold. There was a significant difference in arousal duration determined though behaviour versus *T*_sk_ for thresholds from 13 to 19°C (all *P*<0.05) but there was no difference for *T*_sk_ thresholds from 20 to 27°C (all *P*>0.05). Therefore, as a *T*_sk_ threshold of 18–27°C reliably predicted arousal frequency, and a *T*_sk_ threshold of 20–27°C reliably predicted arousal duration, we chose a *T*_sk_ threshold of 20°C for subsequent analysis.

#### Behavioural data

We relied on the continuous recording from the IR camera system throughout the 110 day hibernation period to quantify arousal frequency and duration after confirming that video reliably recorded the start and end of arousals using *T*_sk_ data. During torpor, all bats in both humidity treatments always huddled in one large cluster per cage and subtle movements associated with grooming or shivering by bats in the huddle and locomotion were easily observed in the video recordings. We defined an arousal for a given bat as determined by the start and end of any movement recorded. Defining the beginning of arousals was straightforward because for 80 arousals (out of a total of 90 based on behavioural data), every time a bat was observed moving, its *T*_sk_ rose above the 20°C *T*_sk_ threshold for at least two consecutive *T*_sk_ readings. For six arousals (out of a total of 90 based on behavioural data), bats showed movement without an increase of *T*_sk_ above the 20°C threshold for two or more readings. There was still an increase in *T*_sk_ (maximum *T*_sk_=11.3–18.6°C for at least one reading of the iButton). For seven arousals (out of the 87 based on the 20°C *T*_sk_ threshold), bats were inactive despite *T*_sk_ increasing above the 20°C threshold for more than two iButton readings but these *T*_sk_ increases were moderate (i.e. *T*_sk_ to a maximum of 23.2°C). In all instances, almost all other bats in the huddle of 10 individuals were moving so the increase in the bats' *T*_sk_ was likely an artifact of heat produced by surrounding normothermic bats. However, for a total of 80 arousals by 18 bats during 29–31 days of hibernation, movement reliably predicted an increase in *T*_sk_ above 20°C for at least two readings, confirming the reliability of video observations to identify arousal onset.

Defining the end of arousals was more difficult because sometimes bats were inactive in the video before re-entering torpor despite defending a high *T*_sk_, or because other bats arousing in the huddle caused heating artifacts. To systematically define a movement threshold marking the end of arousals, we visually compared *T*_sk_ data with the behavioural observations. For 76 arousals determined through behaviour (out of a total of 90 arousals), if a bat remained motionless for more than 1.5 h, its *T*_sk_ fell below the 20°C threshold. In two instances, bats were inactive for approximately 2 h (i.e. two arousals based on our behavioural definition) with no associated decrease in *T*_sk_ below the 20°C threshold (i.e. one arousal based on *T*_sk_ threshold, despite the two activity bouts). In two other instances, bats became active after approximately 2 h (i.e. two arousals based on our behavioural definition); however, there was a decrease in *T*_sk_ below the threshold and no subsequent rewarming associated with this behaviour (i.e. one arousal based on the *T*_sk_ 20°C threshold).

If bats remained inactive for less than 1.5 h after movement was first detected, their *T*_sk_ always remained higher than the *T*_sk_ threshold of 20°C. On two occasions, there was <1.5 h between bouts of movement by bats but *T*_sk_ was continually declining during these events as the bats were returning to torpor, so we considered this as one arousal. We then defined the end of an arousal as the time at which a previously active bat had been motionless for >1.5 h. If a bat exhibited movement after this 1.5 h period, we counted that bout of movement as a new arousal.

Overall, behavioural analysis of bats consistently predicted the frequency and duration of arousals that were also identified by a 20°C *T*_sk_ threshold. The mean duration of arousals quantified by a movement threshold (2.01±0.14 h) and a 20°C *T*_sk_ threshold (2.42±0.13 h) did not differ (*P*=0.17; Dunnett's *post hoc* test) and the total arousals quantified by the movement threshold (*n*=90) was almost identical to that for the 20°C *T*_sk_ threshold (*n*=87).

### Statistical analyses

We removed all bats from hibernation while they were still torpid, so we omitted the last torpor bout for all bats from our analyses. We tested for an effect of humidity treatment and initial body mass on arousal duration (square-root transformed to achieve normality of residuals) using a linear mixed model (R package ‘nlme’; https://CRAN.R-project.org/package=nlme) including Bat ID as a random effect. We then used a second linear mixed model with torpor bout duration (square-root transformed to achieve normality of residuals) as the response variable, and humidity treatment and initial body mass as predictors along with Bat ID included as a random effect. We calculated the repeatability (*R*) of arousal duration and torpor bout duration from the linear mixed model as *V*_I_/(*V*_I_*+V*_Res_), where *V*_I_ is individual variance and *V*_Res_ is the residual variance. Repeatability measures the extent of consistent individual differences (i.e. the proportion of the total variance from repeated measures of a trait that is due to among-individual differences). We tested the significance of the Bat ID random effect using a likelihood ratio test (i.e. −2 times the difference in log-likelihoods between the models with and without the Bat ID random effect, estimated using maximum likelihood, tested against a chi-square distribution) with d.f.=1.

We used a generalized linear model (GLM) (R package ‘MASS’; Venables and Ripley, 2002) with a Poisson distribution to compare the total arousals per bat between the two humidity treatments, with initial body mass as a predictor variable. We then quantified the total drinking frequency per bat as the number of visits to the water dish during which a bat was observed drinking. We compared total drinking frequency between the humidity treatments, including initial body mass as a predictor variable, using a GLM with a Poisson distribution. Additionally, we used a GLM with a binomial distribution to compare the proportions of arousals per bat that included at least one bout of drinking between humidity treatments, again including initial mass as a predictor variable. The dependent variable for this analysis was a composite of the number of arousals with drinking and the number of arousals without drinking.

**Fig. 2.**
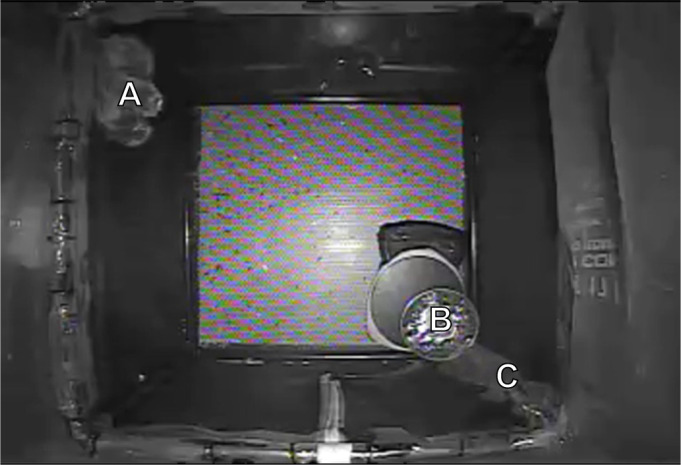
**View from the top of the cage inside an incubator during hibernation.** The image shows a huddle of big brown bats (*n*=10; A), the water dish (with aquarium rocks; B), and the tubing (C) that exits the mesh enclosure to the outside of the incubator for refilling of the water dish. All bats roosted in one huddle throughout hibernation.

Bats in both humidity treatments always huddled in one large cluster per cage during torpor. All bats aroused either singly or in a group cascade (i.e. bat arousals overlapped in time). Therefore, we were able to quantify the density of huddles of bats between each treatment using images (resolution 352×238 pixels) from the IR video recordings in each cage (DW-Pivot Hybrid Central Monitoring Software). Cages in both humidity treatments were identical in size with overhead cameras positioned the same distance from the top of mesh cages where bats roosted, which allowed for a direct comparison. For each photo, we ensured that all bats had been motionless for at least 1 h (for example, see Fig. 2) to ensure that huddle size reflected groups of bats that were torpid or returning to steady-state torpor.

In total, we obtained 24 photos from the humid treatment, and 27 photos from the dry treatment. Huddle photos were randomized, and files were renamed to ensure that we were blind to both study date and humidity treatment when analysing huddle size. We outlined huddles in each photo using ImageJ (v.1.51, National Institutes of Health, Bethesda, MD, USA) and then calculated a huddle size index (HSI) as the percentage of total image area in pixels occupied by the huddle of bats. To ensure measurements were comparable between treatments, we also outlined the water dish in 30 randomized photos (15 from each incubator) and confirmed there was no difference in our size estimation between treatments (Welch's two sample *t*-test, *t*=1.76, d.f.=25.72, *P*=0.09). To confirm that assessment of HSI was repeatable, a second observer, who was also blind to both study date and humidity treatment, repeated huddle-size estimation using the same images. There was no difference in our assessments (Welch's two-sample *t*-test, *t*=−0.38, d.f.=91.3, *P*=0.71) and huddle sizes measured by the two observers were highly correlated (*r*=0.85, *P*<0.001). We then used our measurements to compare the HSI between humidity treatments with a GLM using a quasi-Poisson distribution.

We calculated effect size as Cohen's *d* (R package ‘effsize’; https://CRAN.R-project.org/package=effsize) to compare the magnitude of treatment effects for total arousals per bat, total drinking frequency per bat, proportion of arousals per bat that contained a drinking bout, and HSI between the two humidity treatments (small effect: *d*=0.2; medium effect: *d*=0.5; large effect: *d*=0.8; Cohen, 1988).

We used Welch's *t*-test to ensure that the initial body mass of bats did not differ between the two experimental treatments (*t*=−0.15, d.f.=11.22, *P*=0.88). We then calculated the change in body mass for individual bats from the start to the end of the experiment and used a GLM to test for an effect of humidity treatment on total loss of body mass. These data were right skewed, so we used a gamma error distribution with initial body mass, humidity treatment, total drinking bouts per bat, and total arousals per bat as predictor variables.

All statistical analyses were conducted in R v.4.2.2 (http://www.R-project.org/) using RStudio (v.2022.07.01) with graphs produced using ‘ggplot2’ (Wickham, 2016) in RStudio. For all statistical tests, significance was assessed at alpha <0.05, and values are reported as means±s.e.m. and samples as *n*=number of measurements.

## RESULTS

**Fig. 3.**
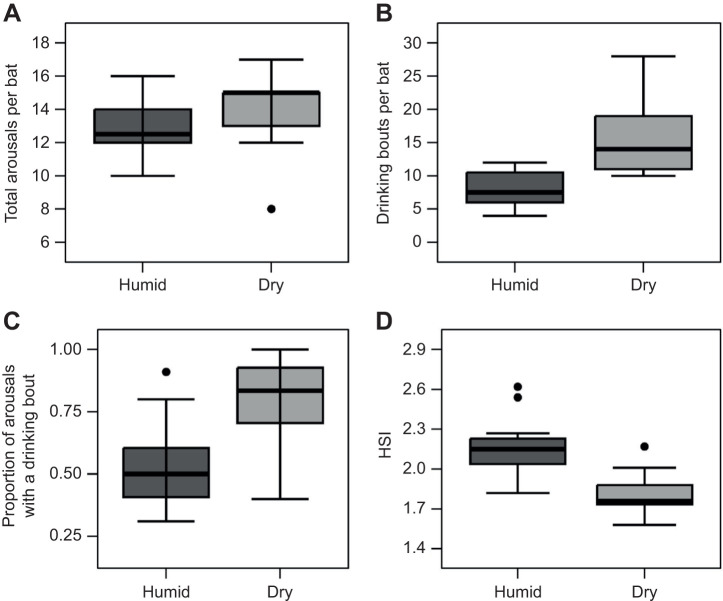
**Arousals, drinking bouts and huddle size for big brown bats in the humid and dry treatments over the 110** **day study period.** (A) Boxplot of total arousals per big brown bat for bats in the humid treatment (*n*=10) and dry treatment (*n*=9). (B) Boxplots of the total drinking bouts per bat for bats in the humid treatment (*n*=10) and dry treatment (*n*=9). (C) Boxplots of the proportion of arousals per bat with a drinking bout for bats in the humid treatment (*n*=10) and dry treatment (*n*=9). (D) Boxplots for the huddle size index (HSI; i.e. percentage of the total image area) of huddling big brown bats from the humid treatment (*n*=24) and dry treatment (*n*=27). For all boxplots, the median is represented by a solid horizontal line, and the top and bottom of each box represent the 25th and 75th percentiles, respectively. The upper whisker represents the maximum value within 1.5 times the interquartile range above the 75th percentile, whereas the lower whisker represents the minimum value of data within 1.5 times the interquartile range below the 25th percentile. Points that are outside the whisker range are included on the plot.

We recorded 129 arousals and 119 torpor bouts from 10 big brown bats in the humid treatment and 124 arousals and 115 torpor bouts from nine bats in the dry treatment. The discrepancy between the number of arousal and torpor bouts is a result of the removal of torpid bats at the end of the experiment. There was no effect of humidity treatment on total arousals per bat (Fig. 3A; GLM, *P*=0.60, Cohen's *d*=0.39) or initial body mass (*P*=0.42) but bats in the dry treatment had shorter arousal durations (106±72 min) than bats in the humid treatment (134±82 min) (Table 1; *P*=0.01). There was no effect of humidity treatment or initial body mass on the torpor bout duration of all bats (Table 2). Both arousal (*R=*3.4×10^8^, χ^2^=5.06, d.f.=1, *P*=0.02) and torpor duration were repeatable (*R=*5.3×10^9^, χ^2^=4.48, d.f.=1, *P*=0.03). Bats in the humid treatment had torpor bout durations of 8.3±0.38 days versus 7.9±0.46 days for bats in the dry treatment. The total number of drinking bouts per bat was 52% higher in the dry treatment than in the humid treatment and had a greater range of variability (Fig. 3B; *P*<0.001, Cohen's *d*=1.67). A positive trend for an effect of initial body mass on drinking bouts per bat approached significance (*P*=0.053) but the fit of the relationship was very weak (linear regression; *r*^2^=0.037). Bats in the dry treatment had a higher proportion of arousals that contained drinking bouts compared with bats in the humid treatment (Fig. 3C; GLM, *P*<0.001, Cohens' *d*=1.32), again with no effect of initial body mass (*P*=0.16). During the first 31 days when iButtons were recording *T*_sk_, drinking behaviour always occurred at relatively warm *T*_sk_ for all bats. Bats in the humid treatment drank at *T*_sk_=28.4±0.58°C and bats in the dry treatment drank at *T*_sk_=29.7±0.28°C (see Figs S1 and S2 for Tsk traces).

**Table 1.**
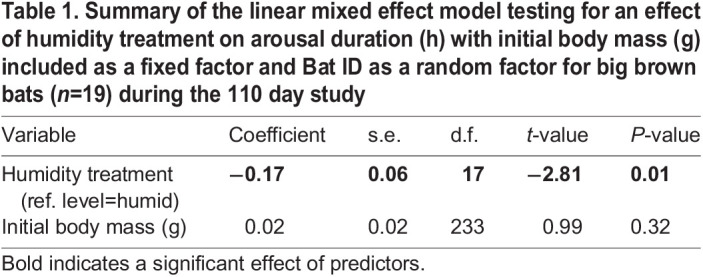
**Summary of the linear mixed effect model testing for an effect of humidity treatment on arousal duration (h) with initial body mass (g) included as a fixed factor and Bat ID as a random factor for big brown bats (*n*=19) during the 110** **day study**

**Table 2.**
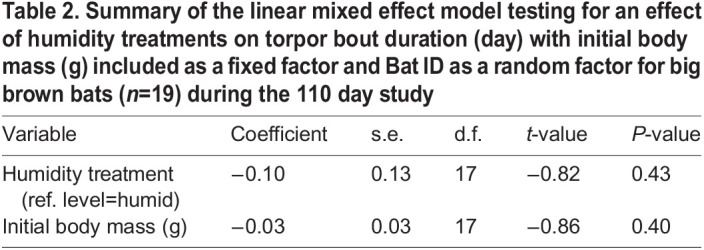
Summary of the linear mixed effect model testing for an effect of humidity treatments on torpor bout duration (day) with initial body mass (g) included as a fixed factor and Bat ID as a random factor for big brown bats (*n*=19) during the 110 day study

The HSI was dramatically smaller (Cohen's *d*=2.12) for bats in the dry treatment (HSI=1.81±0.12) than for bats in the humid treatment (HSI=2.14±0.04) (Fig. 3D; GLM, *P*<0.001), indicating that bats formed a more compact huddle in the dry treatment.

**Fig. 4.**
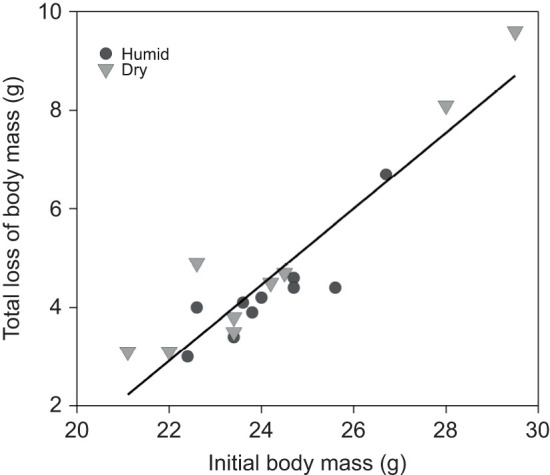
**Relationship between the initial body mass and total loss of body mass of hibernating big brown bats from the humid and dry treatments over the 110** **day experimental period.** The data showed a positive relationship between the two variables for bats in both the humid (*n*=10) and dry (*n*=9) treatments.

There was no effect of total arousals or total drinking bouts per bat, or humidity treatment on total loss of body mass throughout hibernation (all *P*>0.05). However, there was a significant effect of initial body mass on the total loss of body mass (Fig. 4; GLM, *P*<0.001), with the heaviest bats losing the most body mass over the 110 day study period.

## DISCUSSION

Our results support the hypothesis that female big brown bats adjust huddling and drinking behaviour to maintain similar patterns of arousal, and therefore energy expenditure, in conditions of varying humidity. In our study, bats hibernated for 110 days in either humid (98% RH at 8°C) or dry (50% RH at 8°C) conditions, which represented the range of humidity that big brown bats experience in the wild (Klüg-Baerwald and Brigham, 2017). Consistent with our predictions, there was no difference in either the frequency of arousals and torpor bouts, or the duration of torpor bouts, between bats from the humidity treatments. Previous studies have proposed that dehydration induces arousals, and hibernators will return to a normothermic *T*_b_ to drink and restore normal water balance (Ben-Hamo et al., 2013; Thomas and Geiser, 1997). Arousals, however, are energetically expensive, and bats need to budget the high energetic costs of arousals to drink against the physiological costs resulting from dehydration while remaining torpid (Boyles et al., 2020; Humphries et al., 2003). Bats in the dry treatment did not differ in arousal frequency and torpor duration, presumably because of their ability to conserve water via huddling and their increased water intake during arousals. Kallen (1964) hypothesized that EWL equivalent to 4.2% body mass may be a critical threshold for arousal in little brown bats and, more recently, Klüg-Baerwald et al. (2022) provided evidence that free-ranging big brown bats hibernating in dry conditions become dehydrated as hibernation progresses. However, for our study, while bats in the dry treatment may have lost water, they likely remained below the 4.2% threshold for water loss throughout the experiment, which may have prevented the need for more frequent arousals and shorter torpor bouts.

Consistent with our prediction, bats in the dry treatment drank at a greater rate and used a greater proportion of their arousals for drinking compared with bats in the humid treatment. For inactive hibernators, some water can be produced endogenously through metabolic processes during torpor bouts, but this does not fully compensate for EWL over hibernation (Thomas and Cloutier, 1992; Thomas and Geiser, 1997). Thus, bats in the humid treatment still needed to arouse and drink, but at a lower rate than bats in the dry treatment. Conversely, bats in the dry treatment likely experienced higher rates of EWL and had to drink more to restore water balance, but not at a rate that affected arousal frequency, possibly because of their ability to reduce EWL by forming a more compact huddle. Additionally, there was more variability in drinking bouts per bat in the dry treatment (range 10–28) compared with the humid treatment (range 4–12). This increased variability for bats exposed to a dehydrating environment could reflect differences in huddle position of individual bats. Bats located on the periphery of a huddle will have more exposed surface area, causing higher rates of EWL. Thus, these bats would need to drink more to replenish water balance. If individual bats are consistent in their huddle positions, this could lead to greater between-individual variability in the need to drink by different bats, especially in the dehydrating environment of our low humidity treatment. We were unable to identify positions of different individuals, but it would be interesting for future studies to examine individual positions in a huddle and effects of huddle position on relative rates of water loss. We found no effect of total drinking bouts per bat on the total mass loss for bats and, except for a weak trend relating initial body mass and total drinking bouts per bat, there was no clear effect of initial body mass on drinking behaviour. This suggests that bats did not adjust metabolic water production in response to environmental humidity or their available energy stores. Instead, changes in huddling appeared to exert more influence on water balance.

In the beginning of the experiment, bats in both humidity treatments drank only at a normothermic *T*_sk_ (i.e. ∼29°C). This suggests that bats need to use a large amount of energy to rewarm to normothermia and drink as opposed to using ‘cold arousals’ which have been observed in other bat species (Blažek et al., 2019; Mayberry et al., 2018). In the wild, bats roosting in humid conditions may be able to drink condensation that has collected on the walls of caves or on their fur (Davis, 1970). However, there was no condensation on the mesh cages in the humid treatment, so bats in both treatments, which all roosted in the top corner of their cages, needed to crawl to the water dish to drink. Thus, all torpid bats in both treatments needed to rewarm to normothermia to crawl to the water dish to restore water balance. For big brown bats that roost in dry conditions in the wild, these energetic costs are higher because, in addition to rewarming to normothermia, they would have to fly to a water source, potentially outside the hibernaculum (Klüg-Baerwald et al., 2022; Lausen and Barclay, 2002). Ingesting snow or near-freezing water near a cave would also result in thermoregulatory costs, particularly for small-bodied mammals such as bats, and could further constrain bats energetically (Cooper and Withers, 2014). Flight is energetically expensive with MRs 15–16 times higher than at rest (Speakman and Thomas, 2003) and rates of EWL are elevated during flight (Studier, 1970). Combined with the added costs of thermoregulation, the energetic cost of acquiring water for bats in the wild might be remarkably high, which could explain why big brown bats appear capable of using huddling to reduce the need to drink.

Consistent with our prediction, big brown bats in the dry treatment huddled in a more compact huddle during torpor than bats in the humid treatment. This behaviour may have allowed bats in the dry treatment to reduce their surface area exposed to the dehydrating environment. Boratyński et al. (2015) showed that huddling behaviour in Natterer's bats reduced EWL by approximately 30%, compared with bats roosting alone, and this reduction in EWL was most likely due to a reduction in the exposed surface area. However, to date, no studies have explicitly measured the direct effect of variation in huddle density (and thus the degree of exposed surface) on the rate of EWL. Instead, previous studies have measured the effect on huddle density and exposed surface area on MR. For example, Gilbert et al. (2008) showed that, compared with loosely huddled emperor penguins (*Aptenodytes fosteri*), tightly huddled penguins experienced a 38% reduction in MR, and 2/3 of the reduction was attributed to a decrease in exposed surface area to the cold environment (1/3 was attributed to a warmer microclimate within the huddle). We did not observe differences in loss of body mass, and thus energy expenditure, between the humid treatment and dry treatment. Therefore, we hypothesize that the main function of increasing huddle density (and thus decreasing exposed surface area) was not to decrease heat loss to the cold environment, but rather to reduce the rates of EWL and conserve water.

Overall, a decrease in cutaneous EWL, through an increase in huddle density or a decrease in exposed surface area, coupled with a decrease in cutaneous and respiratory EWL, through a decrease in arousal duration, could contribute to an overall decrease in total EWL. Therefore, huddling big brown bats in dry conditions appear to make adaptive behavioural adjustments that reduce EWL which would, in turn, reduce arousal frequency and energy expenditure.

Although humidity did not have a direct effect on total loss of body mass throughout hibernation, bats that started with the highest initial body mass experienced the greatest decline over the study. Total loss of body mass ranged from 3.0 g (initial body mass 22.4 g) to 9.6 g (initial body mass 29.5 g). In a previous study, Kuhl's pipistrelle (*Pipistrellus kuhlii*) that hibernated under dry conditions showed higher arousal frequencies and greater mass loss (Ben-Hamo et al., 2013), which indicates that, for some bats, dry conditions cause increased energy expenditure. We did not find an effect of initial body mass on torpor bout duration, or an effect of the total arousals per bat on total loss of body mass. Therefore, the relationship between initial body mass and decrease in body mass of hibernating bats cannot be attributed to changes in arousal or torpor bouts. Previous studies of hibernating mammals have shown that individuals with larger fat stores are not constrained by energy availability and have more flexibility in arousal expression (Bieber et al., 2014). Mammals that have smaller energy stores express longer and deeper torpor bouts, presumably to reduce the use of energetically expensive arousals (Humphries et al., 2003), but they still need to arouse at some interval. In our study, bats with larger fat stores did not express an increase in arousal frequency (or a decrease in torpor bout duration). This indicates that the variation of lost body mass cannot be attributed to balancing energetically expensive arousals with prolonged torpor. In the context of disease, previous studies in European bats (*Myotis myotis*; Fritze et al., 2021) and little brown bats in North America (Cheng et al., 2018) provide evidence that bats with larger initial fat reserves have lower morbidity and mortality rates from WNS, possibly because of their ability to fuel a great frequency of arousals. Conversely, however, surviving populations of little brown bats show less frequent arousals (and thus longer torpor bouts) (Lilley et al., 2016). While we did not study WNS-affected bats, it would be useful for future studies to assess the effect of fat reserves on morbidity and mortality, and possible WNS resistance in big brown bats.

Social thermoregulation and huddling can be beneficial for individuals, but may also come with costs, which may have contributed to the variation in the total loss of body mass that we observed. In both humidity treatments, bats remained in a single huddle throughout hibernation and aroused from torpor in groups of 2–10 while occasionally, but infrequently, arousing on their own. One possibility is that bats with a larger initial body mass entering hibernation were first to rewarm during these shared arousals and, therefore, experienced higher energetic costs. Conversely, bats in poorer body condition could benefit energetically from passive arousals or ‘arousal cascades’ (Turner et al., 2014). The effect of group synchrony and passive rewarming on energy expenditure has been quantified in other mammals. Alpine marmots (*Marmota marmota*) have a well-defined synchronization of arousals from hibernation, with adults that arouse first experiencing a greater energetic cost, followed by juveniles, which benefit from reduced costs because of passive rewarming (Ruf and Arnold, 2000). We hypothesize that for our study, bats with the greatest total loss of body mass may be individuals that initiated arousal cascades, and thus experienced greater energy expenditure. Bats with the lowest total loss of body mass may have aroused last within the cascade, and experienced a net energetic benefit from other bats in the huddle. Future studies could test this hypothesis by analysing the timing of arousals with the loss of body fat over time. This phenomenon could be influenced by the social dynamics and sex composition of the groups. In our study, all bats were female, and housed together for 28 months prior to the beginning of the study. Little is known about the social dynamics of big brown bats during hibernation but, during the active season, female big brown bats aggregate in maternity colonies to give birth to their pups and conform to a ‘fusion–fission’ colony structure, where bats remain loyal to colony mates but may switch physical roosts (Willis and Brigham, 2004). Female bats that roost communally and give birth to pups can benefit from mutual warming of pups (Trune and Slobodchikoff, 1978) and, thus, overall shared energetic costs. It is possible that roosting communally is a behaviour that persists from the maternity season into hibernation. Thus, future studies could analyse the huddle structure of big brown bats during hibernation and determine whether persistent social dynamics that are established in maternity colonies affect patterns of group hibernation.

In contrast to one of our predictions, bats in the dry treatment had shorter arousals than bats in the humid treatment. Shorter arousals mean bats spend less time normothermic, and thus may decrease respiratory and cutaneous EWL. In Kuhl's pipistrelle, cutaneous EWL was lower during shallow torpor than during normothermia, and respiratory EWL declined as MR decreased (i.e. as bats were becoming torpid) (Muñoz-Garcia et al., 2012). Bats in the dry treatment may have returned to torpor quickly potentially to decrease both cutaneous and respiratory EWL and retain more of the water acquired by drinking. During hibernation, behaviours that contribute to a decrease in energy expenditure (e.g. shorter arousals) may also allow for a decrease in EWL. Energetic costs are high to defend a normothermic *T*_b_ in cold *T*_a_, and by returning to torpor quickly, hibernators can reduce their energy requirements to as low as ∼1% of those needed to defend normothermia (Geiser, 2004). However, we found that the total loss of body mass did not differ between bats in the two humidity treatments, and if shorter arousal durations contributed to a decrease in energy expenditure, the effect was too small for us to detect. Thus, a decrease in arousal duration for bats in the dry treatment may have been more important for decreasing total EWL rather than decreasing energy expenditure.

Our results provide evidence that captive female big brown bats use behavioural mechanisms to conserve water in conditions of variable humidity. In the wild, female bats tend to hibernate conservatively or ‘thriftily’ relative to males because they must use fat reserves left over at the end of hibernation to fuel ovulation and initiate pregnancy in spring (Jonasson and Willis, 2011; Czenze et al., 2017). This thrifty female strategy could also influence water conservation and drive females to increase huddling behaviour, especially in dry conditions. We recommend that future studies experimentally test for differences in huddling behaviour between male and female big brown bats in conditions of varying humidity.

Given the associated link between dehydration and WNS, these behaviours in big brown bats may contribute to their resistance to the disease. To further explore mechanisms of WNS resistance in big brown bats, future studies could analyse the behaviour of *P. destructans*-infected big brown bats in dry conditions. Big brown bats that can roost, and conserve water, in dry environments may benefit from an overall decrease in *P. destructans* growth due to the low ambient humidity, which may further contribute to underlying resistance mechanisms to WNS.

Overall, our results show that female big brown bats adjust huddling and drinking behaviours while maintaining consistent patterns of arousal in conditions of variable humidity. Big brown bats in a dry environment increased huddle density and drinking behaviour, which could allow for the conservation and restoration of water balance. Our results suggest that behavioural flexibility by big brown bats may allow them to tolerate hibernation conditions that are not suitable for other hibernating bat species and could play a key role in their resistance to WNS.

## Supplementary Material

10.1242/jexbio.246699_sup1 Supplementary information

## ECR Spotlight

This article has an associated ECR Spotlight interview with Kristina Muise.
