# Supplementary material for: Hibernating female big brown bats (Eptesicus fuscus) adjust huddling and drinking behaviour, but not arousal frequency, in response to low humidity
Source: J Exp Biol. 2024 Mar 7;227(5):jeb246699. doi: 10.1242/jeb.246699 (PMC10949064; doi:10.1242/jeb.246699)
Supplement: Supplementary information [file jexbio-227-246699-s1.pdf]

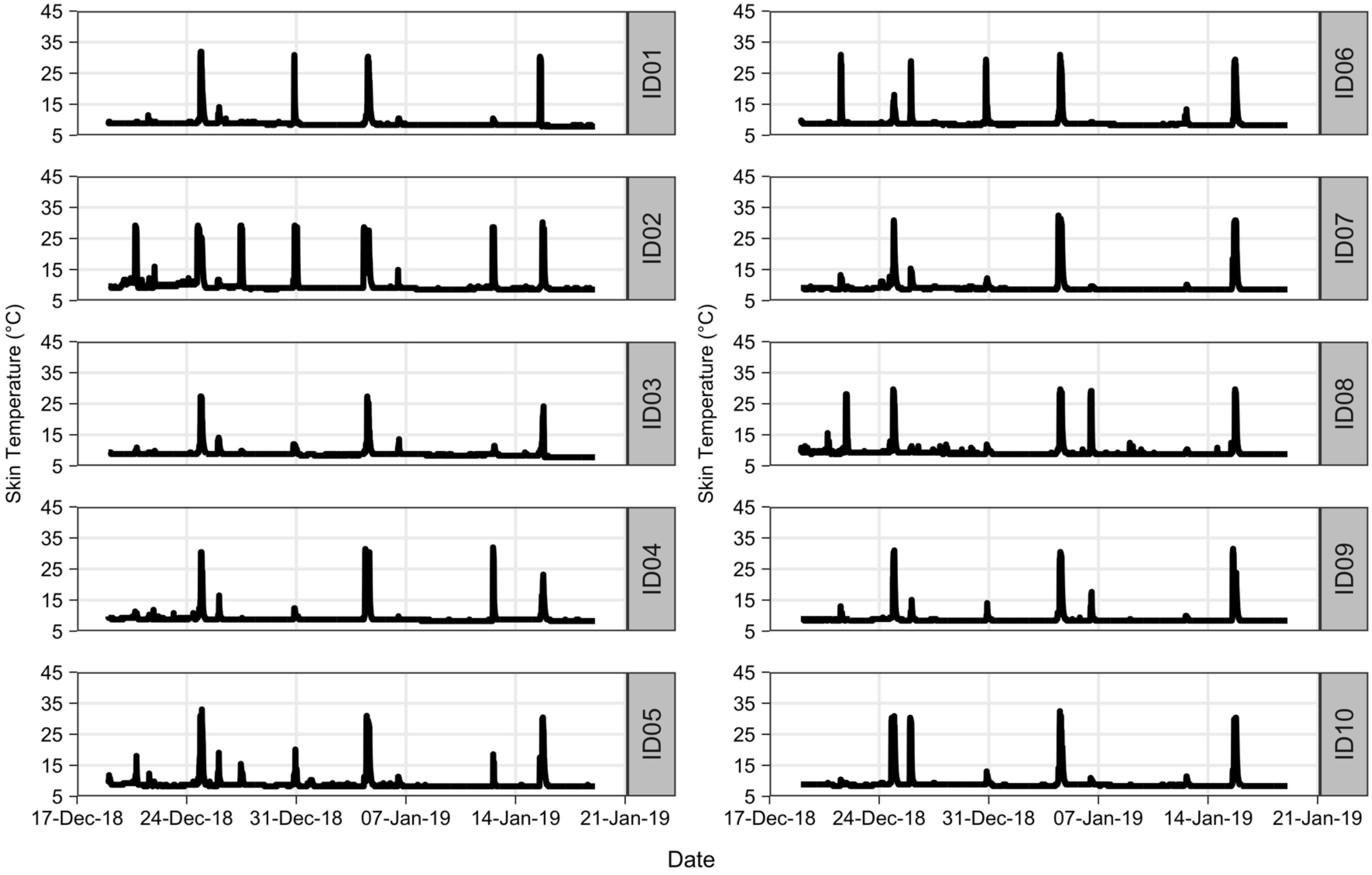

Fig. S1. Skin temperature traces (°C) of hibernating big brown bats (*Eptesicus fuscus*, n = 10) from the *humid treatment* from 18 December 2018 to 19 January 2019 (31 days of the 110-day study period). Bat ID is indicated on the right of each individual graph.

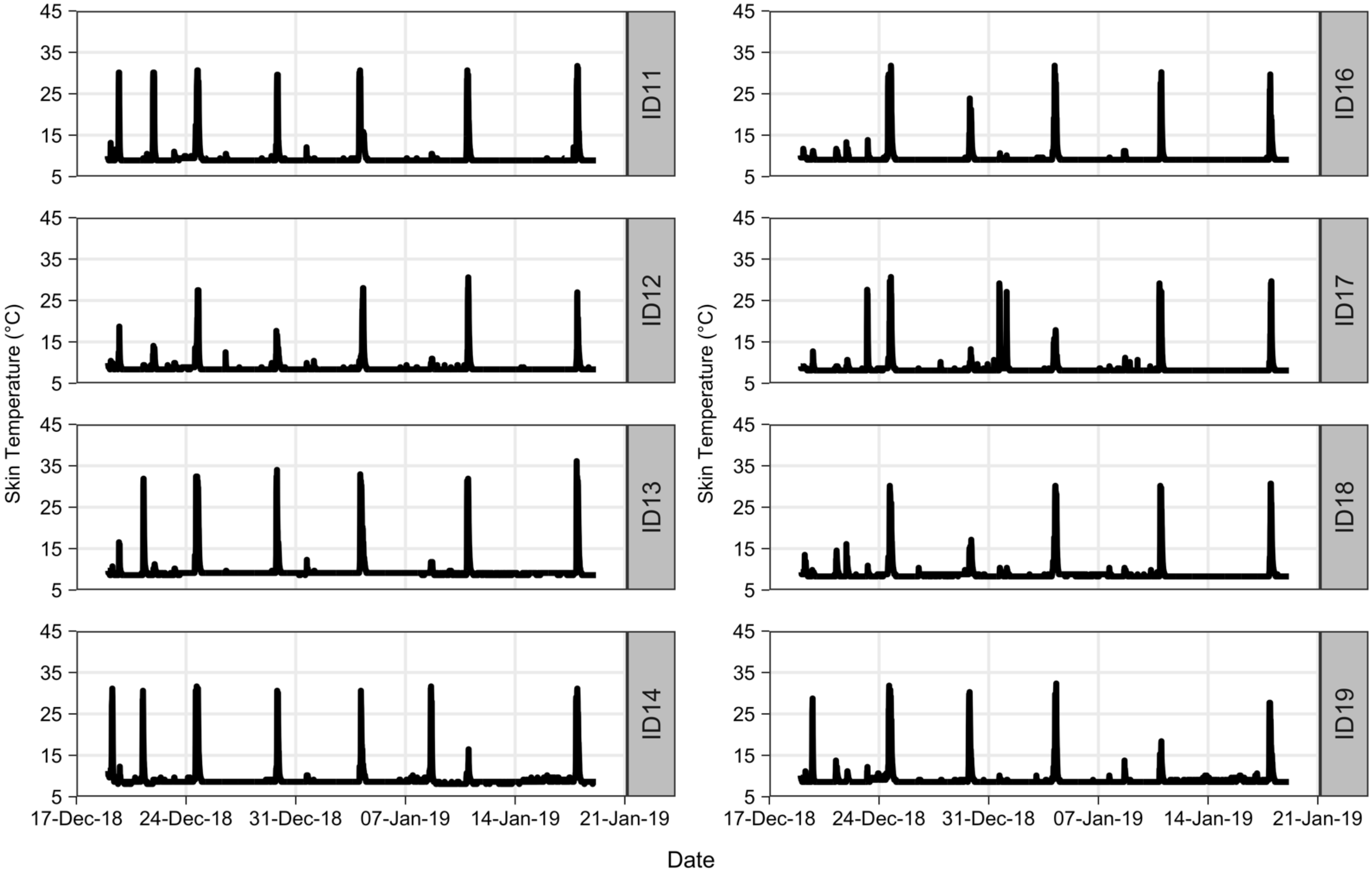

Fig. S2. Skin temperature traces (°C) of hibernating big brown bats (*Eptesicus fuscus*, n = 8) from the *dry treatment* from 18 December 2018 to 19 January 2019 (31 days of the 110-day study period). Bat ID is indicated on the right of each individual graph.
